# Supplementary figures and images for: RNA methyltransferase CMTR-1 inhibition activates a GATA transcription factor-mediated protective immune response
Source: PLoS Pathog. 2026 Jun 24;22(6):e1014375. doi: 10.1371/journal.ppat.1014375 (PMC13318185; doi:10.1371/journal.ppat.1014375)

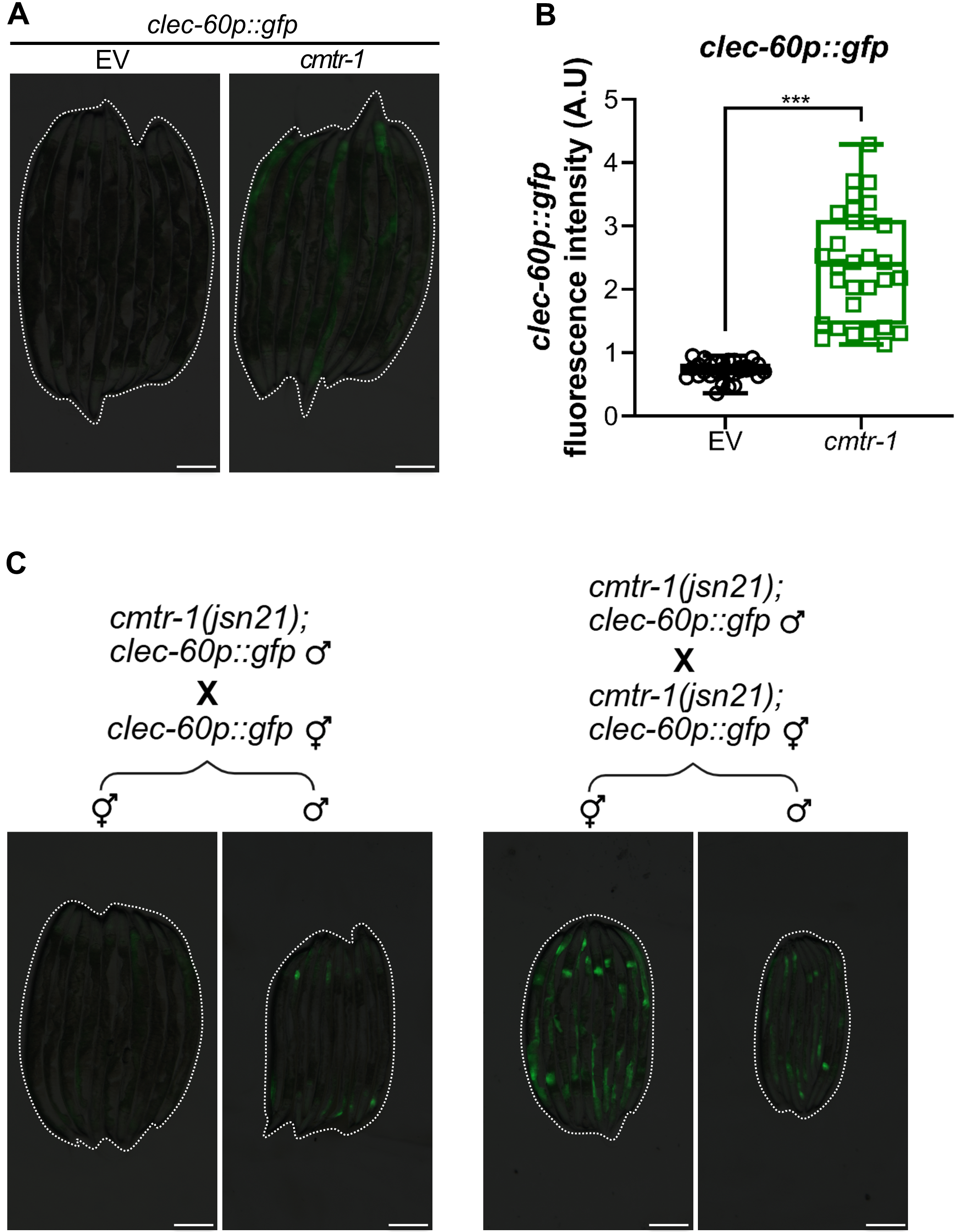

Supplement: S1 Fig — (A) Representative fluorescence images of clec-60p::gfp worms exposed to empty vector (EV) and cmtr-1 RNAi. Dotted outlines indicate worm positions. Scale bar = 200 μm. (B) Quantification of GFP levels of clec-60p::gfp worms exposed to EV and cmtr-1 RNAi. ***p < 0.001 via t-test (n = 30 worms each). (C) Representative fluorescence images of the male and hermaphrodite F1 progeny of the shown genetic crosses. Dotted outlines indicate worm positions. The illustration of the genetic crosses was Created in BioRender. Singh, J. (2026) https://BioRender.com/smkyg2j. Scale bar = 200 μm. (TIF) [file ppat.1014375.s001.tif]

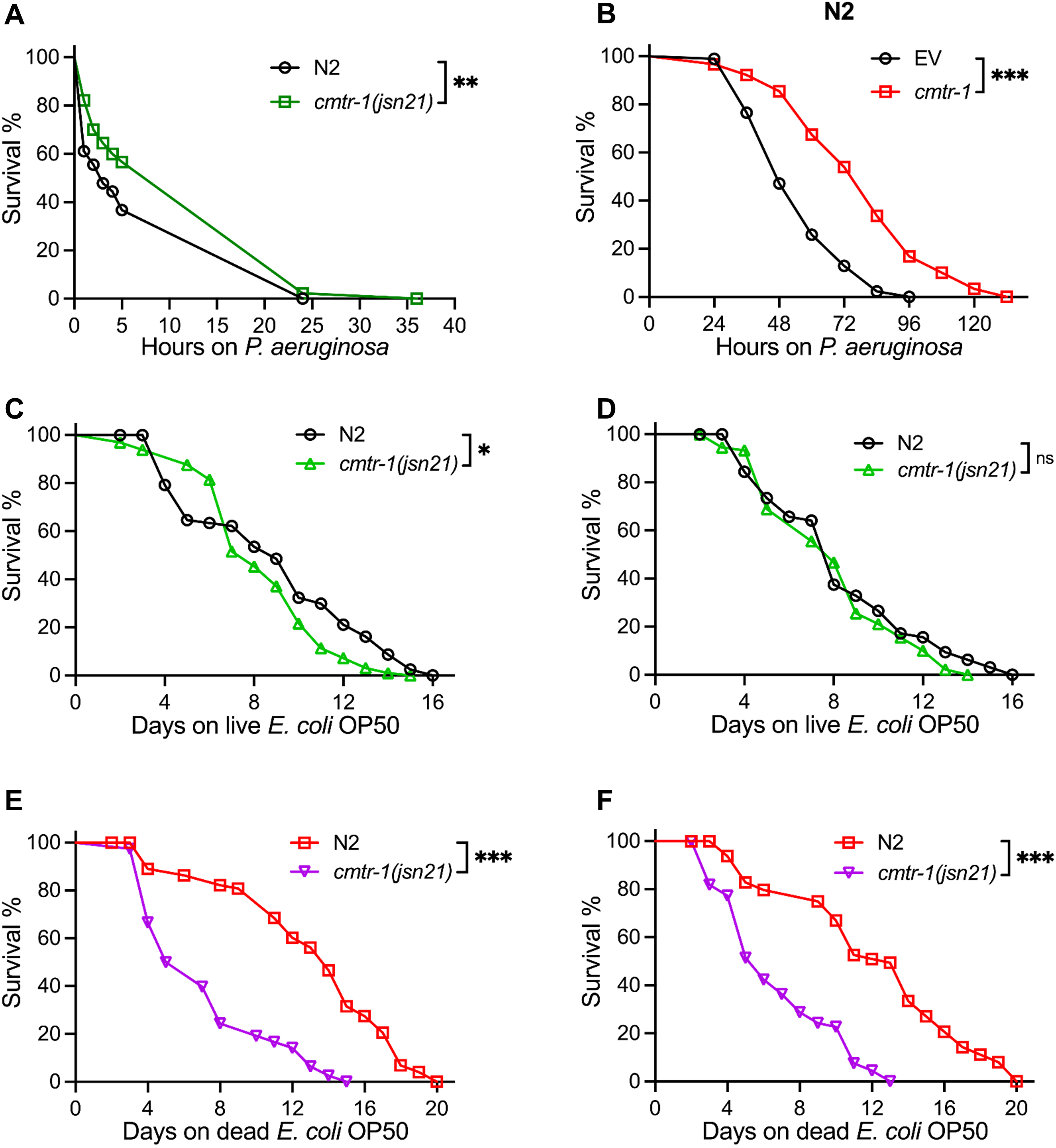

Supplement: S2 Fig — (A) Representative survival plots of N2 and cmtr-1(jsn21) worms on P. aeruginosa PA14 at 25°C under fast-killing assay conditions. **p < 0.01 for the mutant as compared to the control worms (n = 90 per condition). (B) Representative survival plots of N2 worms on P. aeruginosa PA14 at 25°C under slow-killing assay conditions after treatment with the empty vector (EV) control and cmtr-1 RNAi. ***p < 0.001 for cmtr-1 RNAi worms compared to the EV control worms (n = 90 per condition). (C) Representative survival curves of N2 and cmtr-1(jsn21) worms fed on live E. coli OP50 in the absence of FUdR. *p < 0.05 for cmtr-1(jsn21) compared to N2 (n = 92 for N2 and 97 for cmtr-1(jsn21)). (D) Representative survival curves of N2 and cmtr-1(jsn21) worms fed on live E. coli OP50 in the absence of FUdR. The difference between N2 and cmtr-1(jsn21) worms was not statistically significant (n = 93 for N2 and 100 for cmtr-1(jsn21)). Panels (C) and (D) are two independent biological replicates. (E) Representative survival curves of N2 and cmtr-1(jsn21) worms fed on kanamycin-killed E. coli OP50 in the absence of FUdR. ***p < 0.001 for cmtr-1(jsn21) compared to N2 (n = 82 for N2 and 78 for cmtr-1(jsn21)). (F) Representative survival curves of N2 and cmtr-1(jsn21) worms fed on kanamycin-killed E. coli OP50 in the absence of FUdR. ***p < 0.001 for cmtr-1(jsn21) compared to N2 (n = 75 for N2 and 69 for cmtr-1(jsn21)). Panels (E) and (F) are two independent biological replicates. (TIF) [file ppat.1014375.s002.tif]

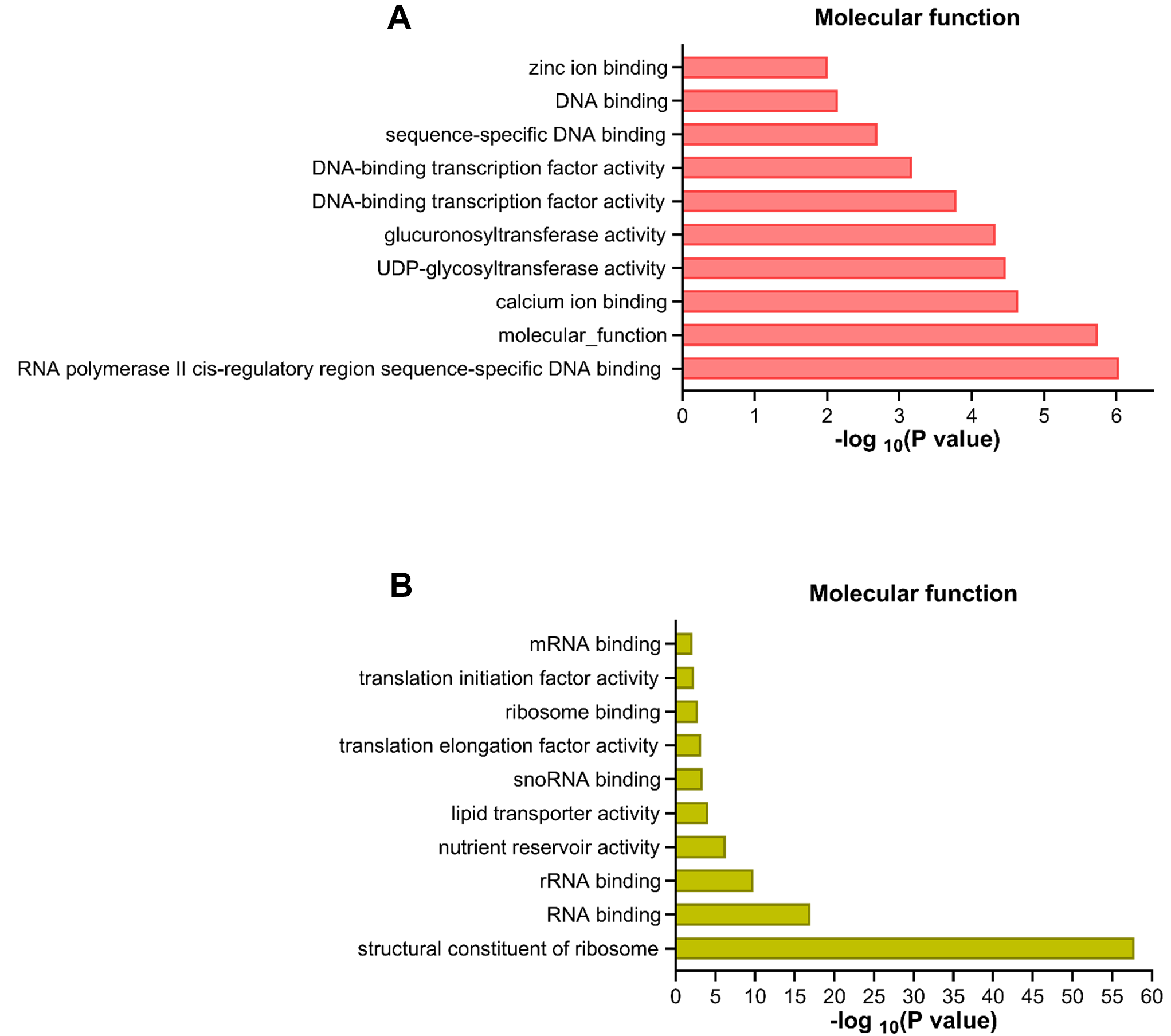

Supplement: S3 Fig — (A)-(B) Gene Ontology (GO) enrichment analysis for molecular functions for upregulated (A) and downregulated (B) genes in cmtr-1(jsn21);clec-60p::gfp worms compared to control clec-60p::gfp worms grown on E. coli OP50. (TIF) [file ppat.1014375.s003.tif]

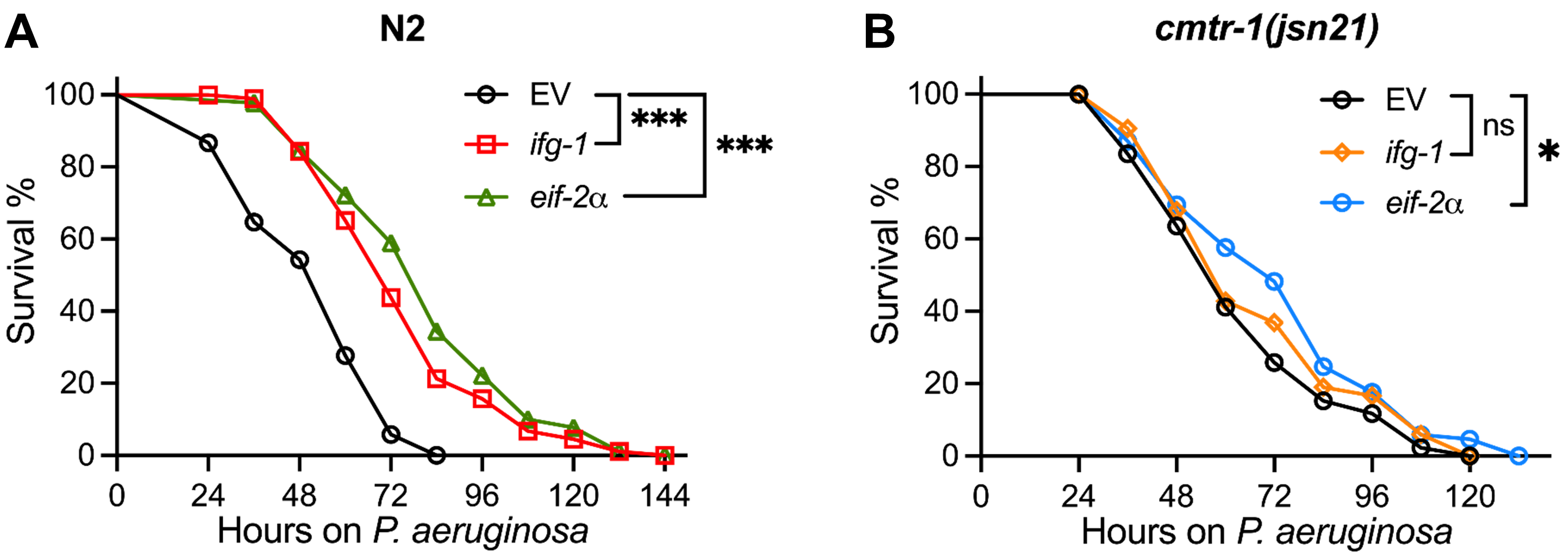

Supplement: S4 Fig — (A) Representative survival plots of N2 worms on P. aeruginosa PA14 at 25°C under slow-killing assay conditions after treatment with the empty vector (EV) control, eif-2α, and ifg-1 RNAi. ***p < 0.001 for eif-2α and ifg-1 RNAi-treated worms compared with EV control worms (n = 90 per condition). (B) Representative survival plots of cmtr-1(jsn21) worms on P. aeruginosa PA14 at 25°C under slow-killing assay conditions after treatment with the EV control, eif-2α, and ifg-1 RNAi. ***p < 0.05 and non-significant (ns) for eif-2α and ifg-1 RNAi-treated worms, respectively, compared with EV control worms (n = 90 per condition). (TIF) [file ppat.1014375.s004.tif]

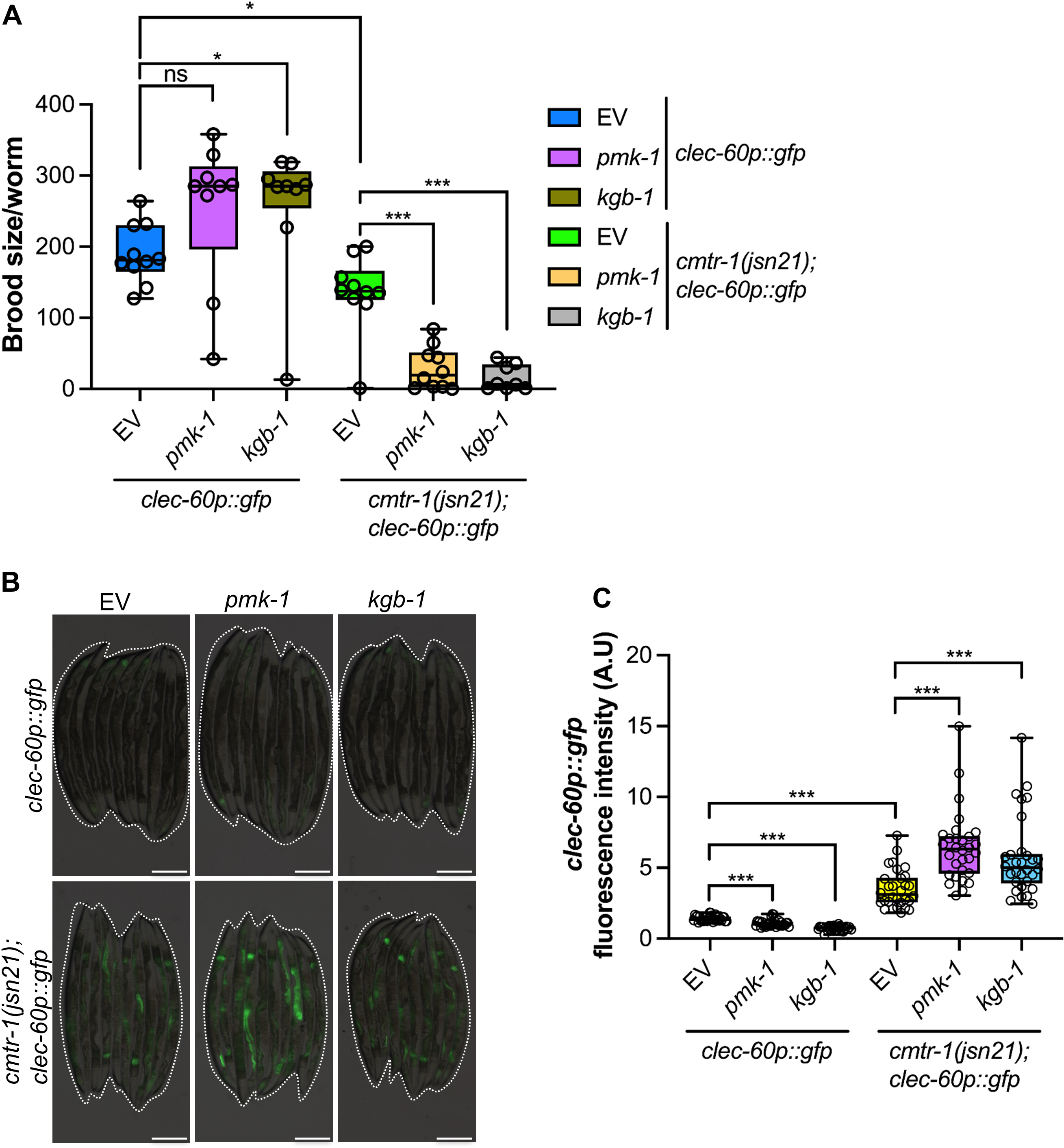

Supplement: S5 Fig — (A) Brood size of clec-60p::gfp and cmtr-1(jsn21);clec-60p::gfp worms grown on the empty vector (EV) control, pmk-1, and kgb-1 RNAi. ***p < 0.001, *p < 0.05, and non-significant (ns) via t-test (n = 8–10 worms each). (B) Representative fluorescence images of clec-60p::gfp and cmtr-1(jsn21);clec-60p::gfp worms grown on EV control, pmk-1, and kgb-1 RNAi. Dotted outlines indicate worm positions. Scale bar = 200 μm. (C) Quantification of GFP levels of clec-60p::gfp and cmtr-1(jsn21);clec-60p::gfp worms grown on EV control, pmk-1, and kgb-1 RNAi. ***p < 0.001 via t-test (n = 30 worms each). (TIF) [file ppat.1014375.s005.tif]

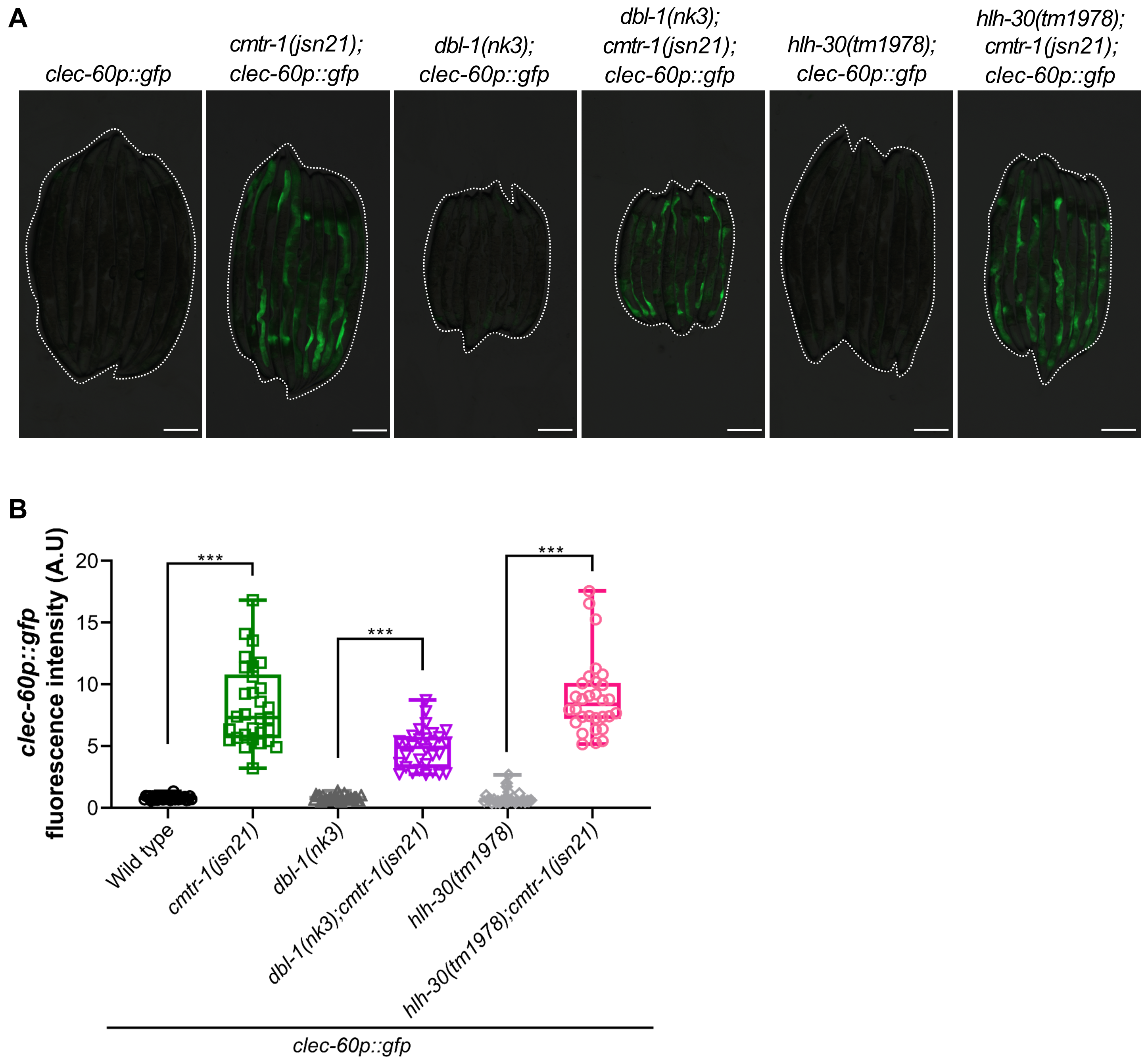

Supplement: S6 Fig — (A) Representative fluorescence images of clec-60p::gfp, cmtr-1(jsn21);clec-60p::gfp, dbl-1(nk3);clec-60p::gfp, dbl-1(nk3);cmtr-1(jsn21);clec-60p::gfp, hlh-30(tm1978);clec-60p::gfp, and hlh-30(tm1978);cmtr-1(jsn21);clec-60p::gfp worms. Dotted outlines indicate worm positions. Scale bar = 200 μm. (B) Quantification of GFP levels of clec-60p::gfp, cmtr-1(jsn21);clec-60p::gfp, dbl-1(nk3);clec-60p::gfp, dbl-1(nk3);cmtr-1(jsn21);clec-60p::gfp, hlh-30(tm1978);clec-60p::gfp, and hlh-30(tm1978);cmtr-1(jsn21);clec-60p::gfp worms. ***p < 0.001 via t-test (n = 29–30 worms each). The controls clec-60p::gfp and cmtr-1(jsn21);clec-60p::gfp are shared with Fig 4B. (TIF) [file ppat.1014375.s006.tif]

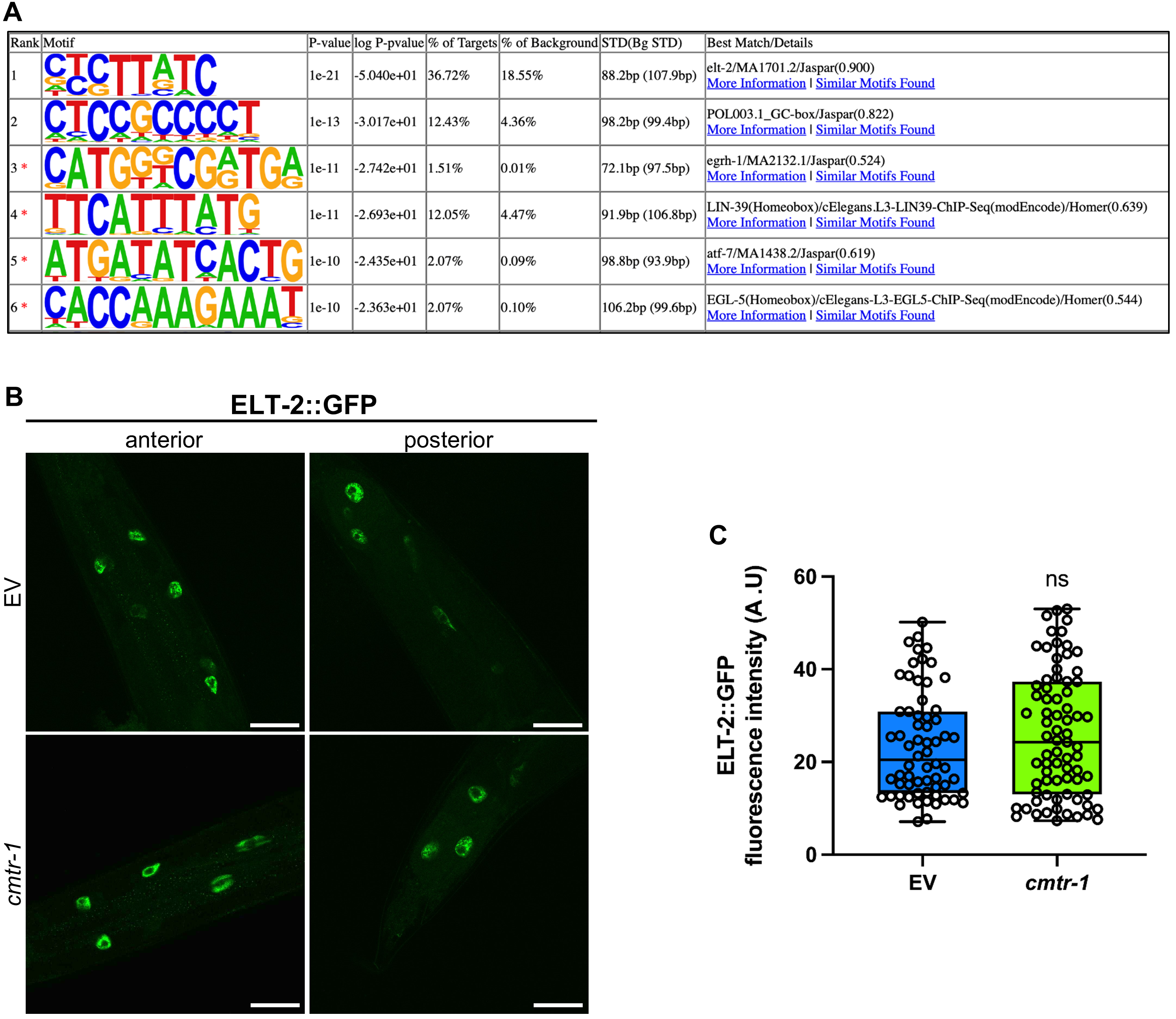

Supplement: S7 Fig — (A) List of the hits obtained for the most enriched binding motifs for the 1217 upregulated genes in cmtr-1(jsn21) worms, ranked in order from lowest to highest p values. (B) Representative confocal fluorescence images of ELT-2::GFP worms grown on empty vector (EV) control and cmtr-1 RNAi. Scale bar = 50 μm. (B) Quantification of GFP levels of ELT-2::GFP worms grown on EV control and cmtr-1 RNAi. Non-significant (ns) via t-test (n = 64 nuclei for EV and 75 nuclei for cmtr-1 RNAi). (TIF) [file ppat.1014375.s007.tif]
