# Supplementary material for: RNA methyltransferase CMTR-1 inhibition activates a GATA transcription factor-mediated protective immune response
Source: PLoS Pathog. 2026 Jun 24;22(6):e1014375. doi: 10.1371/journal.ppat.1014375 (PMC13318185; doi:10.1371/journal.ppat.1014375)
Supplement: Attachment — Submitted filename: Response to Reviewers.pdf [file ppat.1014375.s011.pdf]

We thank the editor and reviewers for their interest in our work and for providing constructive feedback that has substantially improved the quality of our study. We have carefully considered and addressed all the comments raised by the reviewers. Below, we provide a detailed, point-by-point response. Reviewer comments are presented in blue, and our responses are in black. We believe that all concerns have been thoroughly addressed in the revised manuscript.

Reviewers' Comments:

Reviewer's Responses to Questions

Part I - Summary

Please use this section to discuss strengths/weaknesses of study, novelty/significance, general execution and scholarship.

Reviewer #1: The authors found that a forward genetic screen using a clec-60p::gfp reporter identified a hypomorphic allele of the mRNA cap methyltransferase CMTR-1 that activates basal immune signaling in C. elegans. Transcriptomic analysis showed broad induction of innate immune genes in cmtr-1(jsn21) mutants, leading to enhanced resistance to PA14. A transcription factor RNAi screen and bioinformatic analyses identified the intestinal GATA factor ELT-2 as the key regulator of this response, indicating that disruption of CMTR-1–mediated mRNA cap modification triggers a protective immune program.

Overall, this study identifies CMTR-1–mediated mRNA cap modification as a potential regulator of basal immune activation in C. elegans and provides evidence that disruption of this pathway induces a broad innate immune program that enhances resistance to PA14 infection. The genetic screen, transcriptomic analyses, and transcription factor RNAi screen collectively suggest that the intestinal GATA transcription factor ELT-2 plays an important role in mediating this response. However, several conclusions, particularly those regarding translation-independent immune activation and independence from major immune pathways, require additional experimental support. Addressing these concerns through further genetic epistasis analyses and mechanistic experiments would strengthen the authors' claims and clarify the positioning of CMTR-1 within established immune signaling pathways. With these revisions, the study has the potential to provide meaningful insight into how perturbations in mRNA cap modification can act as a signal to activate protective immune responses.
Response: We thank the reviewer for the thoughtful summary of our study and for the constructive and valuable feedback.

Reviewer #2: The authors seek to discover novel regulators of the innate immune response in C. elegans and perform a forward genetic screen to identify mutations that upregulate the immune response gene clec-60. They confirm that G126E missense mutation in the mRNA cap1 methyltransferase cmtr-1 induces clec-60 expression in a recessive manner. Concomitantly, the authors observe that cmtr-1(G126E) confers resistance to PA14 infection and prevents intestinal accumulation of PA14-GFP. cmtr-1 is an essential gene required for the translation of all capped mRNAs, so to understand whether this mutation specifically activates the immune response the authors perform RNA sequencing. They find that the most

prominently upgoing genes are indeed annotated as immune response genes. Using a candidate approach, the authors observe that induction of clec-60 by cmtr-1(G126E) is independent of canonical immune response pathways mediated by ZIP-2, DBL-1, and HLH-30. Finally, the authors identify the transcription factor ELT-2 as being required for the upregulation of clec-60 by cmtr-1(G126E) and the resulting resistance to PA14 infection. Together the findings uncover a novel regulator of the C. elegans immune response and open up interesting future directions such as (1) what is the mechanism for how cmtr-1(G126E) alters gene expression of ELT-2 targets, and (2) are there physiological contexts in which CMTR-1 activates the immune response? In general, the experiments are carefully done and the manuscript is clearly written. However some of their conclusions need rewording or additional support before publication, which I support.
Response: We thank the reviewer for an excellent summary of the study.

Reviewer #3: Here the authors identify a knowledge gap in the field that can be alleviated through systemic perturbations to uncover endogenous pathways that enhance pathogen resistance using clec-60p::gfp reporter expression as a readout. With the use of an EMS screen in C. elegans, they identify a recessive mutation in cmtr-1 causing expression to be high without the presences of a pathogen. Through further verification, they find that this loss-of-function mutation enhances resistance to the pathogen Pseudomonas aeruginosa in slow-kill assays, corresponding to reduced intestinal accumulation of PA14. To understand if the cmtr-1lf mutant was exerting a broad innate immunity transcriptional response, they performed RNAseq and found innate immune genes were upregulated while the translation-related genes were downregulated. To determine if the enhanced immunity was due to translation inhibition, they examined clec-60p::gfp expression in zip-2 mutants with the cmtr-1 mutant background and found immune activation occurs independently from ZIP-2. Using GO term enrichment analysis and HOMER motif enrichment, the authors identified the ELT-2 binding motif as enriched, ELT-2 functions downstream of cmtr-1(jsn21) and is required for the enhanced pathogen resistance. Overall, this work provides an interesting and unique perspective in the field of immune activation and pathogenicity.
Response: We thank the reviewer for an excellent summary of our work.

Part II – Major Issues: Key Experiments Required for Acceptance
Please use this section to detail the key new experiments or modifications of existing experiments that should be absolutely required to validate study conclusions.

Generally, there should be no more than 3 such required experiments or major modifications for a "Major Revision" recommendation. If more than 3 experiments are necessary to validate the study conclusions, then you are encouraged to recommend "Reject".

Reviewer #1:
Comment: 1) cmtr-1 mutants promoting PA14 resistance independent of translation inhibition: I think the authors need to analyse further before making such a bold conclusion. Here, the authors only tested the role of ZIP-2 in contributing to PA14 resistance. A better strategy would be to analyse clec-60::gfp or PA14 resistance following RNAi inhibition of protein translation-related genes that are reduced in the cmtr-1 mutants.

Response: We thank the reviewer for this helpful suggestion. To address this point, we examined the survival of *cmtr-1(jsn21)* mutants following knockdown of the translation initiation factors *eif-2α* and *ifg-1*. We have previously shown that inhibition of these initiation factors enhances survival during *P. aeruginosa* infection (PMID: 39347574). Interestingly, knockdown of either *eif-2α* or *ifg-1* did not further increase the survival of *cmtr-1(jsn21)* mutants, suggesting that the enhanced pathogen resistance of *cmtr-1(jsn21)* worms may be associated with reduced translation. In light of these findings, we removed the statement claiming that the increased survival of *cmtr-1(jsn21)* mutants on *P. aeruginosa* is independent of translation inhibition. These results are described on page 7, lines 183-194: "*Because cmtr-1(jsn21) mutants exhibited reduced expression of translation-related genes (Fig 3C), we next investigated whether immune activation in these animals resulted from impaired protein synthesis. Inhibition of translation initiation is known to activate innate immune responses and enhance resistance to P. aeruginosa under slow-killing conditions [7]. Since cmtr-1(jsn21) worms also displayed reduced expression of translation initiation factors (Table S1), we asked whether their increased survival on P. aeruginosa was linked to reduced translation. To test this, we knocked down the translation initiation factors eif-2α and ifg-1 in wild-type and cmtr-1(jsn21) worms and examined survival during P. aeruginosa infection. Consistent with previous findings [7], inhibition of eif-2α and ifg-1 increased the survival of wild-type worms (Fig S4A). However, knockdown of either factor did not further enhance the survival of cmtr-1(jsn21) mutants (Fig S4B), suggesting that the increased pathogen resistance of cmtr-1(jsn21) worms may be associated with reduced translation.*"

Comment: 2) cmtr-1 mutants promoting PA14 resistance independent of major immune pathways.
I am not convinced that the authors carried out enough genetic epistasis experiments to conclude this. Although generating kgb-1 and pmk-1 double mutants with cmtr-1 was not possible, the authors could use RNAi to inhibit kgb-1 or pmk-1 in the cmtr-1 for two generations. Alternatively, they could perform cmtr-1(RNAi) in the kgb-1 and pmk-1 mutants and assess survival on PA14. clec-60 qPCR can also be done.
Response: We thank the reviewer for this valuable suggestion. To address this point, we examined the effects of *pmk-1* and *kgb-1* knockdown in *cmtr-1(jsn21)* worms. Although depletion of either gene caused severe defects in brood size in the *cmtr-1(jsn21)* background, it did not suppress *clec-60* expression. These findings suggest that the enhanced immune activation observed in *cmtr-1(jsn21)* worms is unlikely to be mediated through the PMK-1 or KGB-1 pathways. The results are described on page 8, lines 212-220: "*To circumvent this limitation, we examined the effects of pmk-1 and kgb-1 knockdown by RNAi. Although cmtr-1(jsn21);clec-60p::gfp worms developed on pmk-1 and kgb-1 RNAi, they exhibited either severely reduced brood sizes or complete sterility (Fig S5A). In contrast, knockdown of pmk-1 or kgb-1 did not affect the brood size of clec-60p::gfp control worms. Notably, cmtr-1(jsn21) mutants also displayed a modest reduction in brood size under control conditions, potentially reflecting a fitness trade-off associated with elevated immune activity, as reported previously [38]. Despite the effects on brood size, knockdown of either pmk-1 or kgb-1 failed to suppress clec-60p::gfp induction in cmtr-1(jsn21) mutants (Fig S5B, S5C).*"

Comment: 3) elt-2(RNAi) suppresses the PA14 resistance of cmtr-1 mutants to the level same as elt-2(RNAi) alone. This suggests that elt-2 is required for survivability on PA14

independent of any upstream perturbations. Authors should use ELT-2::GFP and check for nuclear localization in the absence of cmtr-1. This will provide more support for ELT-2 functioning downstream of CMTR-1.

Response: We examined the effect of *cmtr-1* knockdown on ELT-2::GFP localization and intensity. We did not observe any significant change in the nuclear levels of ELT-2::GFP following *cmtr-1* knockdown. The results are described on page 9, lines 243-246: "*We next examined whether cmtr-1 inhibition alters the nuclear abundance of ELT-2. Knockdown of cmtr-1 did not affect the nuclear intensity of ELT-2::GFP (Fig S7B, S7C), suggesting that CMTR-1 inhibition likely modulates ELT-2 activity independently of changes in its nuclear localization or abundance.*"

Reviewer #2:

Comment: 1. In the section beginning on line 164 and the title to Figure 4 the authors claim, "Enhanced immunity in cmtr-1(jsn21) is independent of translation inhibition". I do not think the data is the paper is sufficient to support this statement. The authors do convincingly show that enhanced immunity by cmtr-1 is independent of ZIP-2, but couldn't translation inhibition still be mediating the cmtr-1 effect in a ZIP-2-independent manner? To support this statement the authors would need to inhibit translation through alternate means (there are many drugs and mutations that can do this) and show that this does not induce clec-60 in a zip-2 mutant. In lieu of these experiments, perhaps the authors would like to scale back their claims.

Response: We thank the reviewer for this helpful suggestion. To address this point, we examined the survival of *cmtr-1(jsn21)* mutants following knockdown of the translation initiation factors *eif-2α* and *ifg-1*. We have previously shown that inhibition of these initiation factors enhances survival during *P. aeruginosa* infection (PMID: 39347574). Interestingly, knockdown of either *eif-2α* or *ifg-1* did not further increase the survival of *cmtr-1(jsn21)* mutants, suggesting that the enhanced pathogen resistance of *cmtr-1(jsn21)* worms may be associated with reduced translation. In light of these findings, we removed the statement claiming that the increased survival of *cmtr-1(jsn21)* mutants on *P. aeruginosa* is independent of translation inhibition. These results are described on page 7, lines 183-194: "*Because cmtr-1(jsn21) mutants exhibited reduced expression of translation-related genes (Fig 3C), we next investigated whether immune activation in these animals resulted from impaired protein synthesis. Inhibition of translation initiation is known to activate innate immune responses and enhance resistance to P. aeruginosa under slow-killing conditions [7]. Since cmtr-1(jsn21) worms also displayed reduced expression of translation initiation factors (Table S1), we asked whether their increased survival on P. aeruginosa was linked to reduced translation. To test this, we knocked down the translation initiation factors eif-2α and ifg-1 in wild-type and cmtr-1(jsn21) worms and examined survival during P. aeruginosa infection. Consistent with previous findings [7], inhibition of eif-2α and ifg-1 increased the survival of wild-type worms (Fig S4A). However, knockdown of either factor did not further enhance the survival of cmtr-1(jsn21) mutants (Fig S4B), suggesting that the increased pathogen resistance of cmtr-1(jsn21) worms may be associated with reduced translation.*"

Comment: 2. From genetic epistasis experiments the authors conclude that elt-2 acts downstream of cmtr-1(G126E) to mediate clec-60 expression. This is supported by elt-2 RNAi not affecting the low basal level of immune response genes via qPCR (Fig S4b). However in the more sensitive gfp reporter assay, elt-2 RNAi does lower the basal expression

of clec-60::gfp (Figure 5d, p<0.001). Have the authors tested whether elt-2 is required for clec-60 induction by pathogen? If it is not required, that would support their claims that elt-2 acts downstream of cmtr-1, as opposed to being a general factor required for basal transcription of immune genes. Perhaps the authors would like to leave this open as a possible interpretation of their data.

Response: We thank the reviewer for this suggestion. To address this point, we examined whether the immune genes induced in *cmtr-1(jsn21)* mutants are also activated during *P. aeruginosa* infection. Interestingly, all of the tested genes were similarly induced upon pathogen exposure. Importantly, the infection-induced expression of these genes, including *clec-60*, was fully dependent on ELT-2. These findings indicate that *cmtr-1(jsn21)* mutants and *P. aeruginosa* infection activate an overlapping immune transcriptional program that is regulated by ELT-2. The results are described on page 9, lines 259-270: "*We next asked whether the immune genes upregulated in cmtr-1(jsn21) mutants are also induced during P. aeruginosa infection. Notably, all tested genes that were upregulated in cmtr-1(jsn21) mutants were also induced following P. aeruginosa infection (Fig 6D), suggesting that cmtr-1 inhibition primes components of the immune response normally activated during pathogen infection. Importantly, induction of these genes during P. aeruginosa infection was fully suppressed by elt-2 knockdown (Fig 6D), indicating that ELT-2 regulates these immune genes in response to both pathogen infection and cmtr-1 inhibition. In contrast, elt-2 knockdown did not reduce the basal expression of these genes prior to pathogen exposure (Fig 6D), suggesting that ELT-2 is dispensable for their constitutive expression under non-infectious conditions. Collectively, these findings demonstrated that ELT-2 controls a common immune transcriptional program activated both during P. aeruginosa infection and upon inhibition of CMTR-1.*"

Reviewer #3:

Comment: 1. The effects of pathogens on host physiology can result due to the presence of the microbe or from the toxins that are secreted. In C. elegans, it is common to test pathogen resistance in the presence of fast-kill assays and slow-kill assays. This manuscript only includes slow-kill assays, and this reviewer would recommend strengthening their argument by adding in fast-kill assay data as well. The reviewer acknowledges that the two assays test two different things (death via colonization vs. death from produced toxins) but still thinks this assay should be included.

Response: We thank the reviewer for the suggestion. As suggested by the reviewer, we performed fast-killing assays of *cmtr-1(jsn21)* worms using *P. aeruginosa* PA14. We observed that *cmtr-1(jsn21)* mutants exhibited significantly increased survival compared with N2 worms under fast-killing conditions as well. The results are described on page 5, lines 126-133: "*We next tested whether elevated clec-60p::gfp expression in cmtr-1(jsn21) mutants correlated with protection against P. aeruginosa PA14 infection. P. aeruginosa PA14 employs multiple mechanisms to kill C. elegans [26]. When PA14 is cultured on minimal medium, infection-mediated lethality develops gradually over several days and is therefore termed "slow killing." In contrast, growth of PA14 on high-osmolarity medium results in rapid lethality within a few hours, referred to as "fast killing." We found that cmtr-1(jsn21) mutants exhibited significantly enhanced resistance to P. aeruginosa infection under both fast-killing (Fig S2A) and slow-killing conditions (Fig 2A).*"

Comment: 2. Lifespan assays were performed exclusively with 50 ug/mL FUdR. FUdR has many complex effects on aging phenotypes and many in the field believe that it is necessary to have at least one replicate of lifespan data without FUdR to support findings from the use with FUdR due to possible confounding effects. The authors should perform at least one replicate without FuDR for lifespan assays to confirm that the results are not due to FUdR and are due to the mutation in cmtr-1.
Response: We thank the reviewer for this suggestion. To strengthen the lifespan analysis, we performed two independent biological replicates for lifespan assays on both live and dead *E. coli* OP50 in the absence of FUdR. These experiments consistently showed that *cmtr-1(jsn21)* mutants have a lifespan comparable to wild-type worms on live bacteria, but exhibit reduced survival on dead bacteria. The results are described on page 6, lines 159-163: "*Because FUdR can influence lifespan in a genotype-dependent manner [33], we further examined longevity in the absence of FUdR. Under these conditions, cmtr-1(jsn21) mutants displayed a lifespan comparable to that of wild-type worms when maintained on live bacteria (Fig S2C, S2D), but exhibited significantly reduced survival on dead E. coli OP50 (Fig S2E, S2F).*"

Part III – Minor Issues: Editorial and Data Presentation Modifications
Please use this section for editorial suggestions as well as relatively minor modifications of existing data that would enhance clarity.

Reviewer #1:
Comment: 1) Incidentally, an identical point mutation was independently isolated in a genetic screen for suppressors of hyperoxia sensitivity in gas-1(fc21) mutants (Meisel et al., 2024), where it displayed a dominant inheritance. (Line 103-105) is not useful.
Response: We have removed this sentence.

Comment: 2) Strikingly, while intestine-specific expression of cmtr-1 restored colonization, pan-tissue expression failed to do so (Figure 2D, E). This discrepancy was unexpected because both rescue constructs suppressed the enhanced survival phenotype. (Line 133-135) Can authors do a tissue-specific knockdown (RNAi) using sensitive RNAi strains and determine the tissue specificity of CMTR-1?
Response: From our experiments, it is clear that intestinal expression of CMTR-1 is sufficient to rescue all major phenotypes of *cmtr-1(jsn21)* mutants, including elevated clec-60 expression, enhanced survival on *P. aeruginosa*, and reduced intestinal colonization. However, the discrepancy between the pan-tissue rescue and the intestine-specific rescue with respect to bacterial colonization was unexpected. To address this issue, we have added possible explanations in the revised manuscript. The revised manuscript reads (pages – 5-6, lines – 142-150): "*Notably, intestine-specific expression of cmtr-1 restored intestinal colonization, whereas pan-tissue expression failed to do so (Fig 2D, 2E). This observation was unexpected because both rescue constructs effectively suppressed the enhanced survival phenotype. One possible explanation is that the expression pattern driven by the sur-5 promoter does not fully recapitulate endogenous cmtr-1 expression. Additionally, previous studies have demonstrated that reduced bacterial colonization and enhanced survival are not always directly correlated [7,12,28]. Nevertheless, these findings established that intestinal CMTR-1 is sufficient to restore colonization in cmtr-1(jsn21) mutants.*"

Comment: 3) Can authors please check the clec-60 mRNA levels in wildtype animals following PA14 exposure in control and elt-2(RNAi) to determine the requirement of elt-2 in promoting clec-60 expression?
Response: The data is provided in the new Figure 6D.

Comment: 4) Genotype nomenclature: please remove underscore (-) when writing for genotypes for two different genes. Instead, use a comma for RNAi and semi colon for double mutants.
Response: We thank the reviewer for the feedback. We have made the corrections.

Reviewer #2:
Comment: 3. The authors should include some discussion of the effects G126E mutation may have on CMTR-1 activity. The cmtr-1(G126E) mutation lies in the G-patch domain of CMTR-1 (not the methyltransferase domain), which binds to the RNA helicase DDX-15 and regulates CMTR-1's methyltransferase activity. Interestingly, disruption of the G-patch domain in vitro can either activate or inhibit CMTR-1 activity (see work from labs of Victoria Cowling, Janusz Bujnicki). I don't think it's necessary for the authors to attempt DDX-15 RNAi in their clec-60::gfp strain, but some acknowledgement of the work done on CMTR-1's G-patch domain would add to the scope of the paper and inform on potential mechanisms.
Response: We thank the reviewer for the suggestion. We have added this in the revised manuscript (page – 4, lines – 101-109): "*The isolated allele, cmtr-1(jsn21), contained a single G → A substitution leading to a Glycine (G) to Glutamic acid (E) substitution at residue 126 (Fig 1D, 1E). This mutation lies within the G-patch domain of CMTR-1, which mediates interaction with the RNA helicase DDX-15 [23–25]. Previous studies have shown that this interaction can modulate CMTR-1 methyltransferase activity, either positively or negatively, depending on the context [23,24]. We therefore examined the inheritance pattern of cmtr-1(jsn21) with respect to clec-60p::gfp upregulation and found that the phenotype segregates recessively (Fig S1C), suggesting that the mutation likely reduces CMTR-1 activity to induce clec-60 expression.*"

Reviewer #3:
Comment: 1. Throughout the manuscript and within figures and figure legends the authors switch between the use of wild-type and N2. All mentions of N2 should be replaced with WT to prevent any confusion that may arise, especially with readers not familiar with C. elegans strains.
Response: We have done this because, for some experiments, *clec-60p::gfp* is the wild-type control, while for others, N2 is the wild-type control. To provide information about N2, we have now explicitly stated in the Methods that N2 is the wild-type control. The revised manuscript reads (page – 11, lines – 323-324): "*Bristol N2 hermaphrodites were used as the wild-type control unless otherwise indicated.*"

Comment: 2. Most of the survival plots and lifespan plots don't contain statistical information in the figure (only in the legends). It would be helpful to show stats on the figures as well.

Response: We have done this for all the survival plots.

Comment: 3. Lines 124-129: When discussing improved pathogen resistance, authors should note in the main manuscript text that the assay performed was a slow-kill assay since both slow-kill and fast-kill assays are the standard to perform when measuring pathogen resistance.

Response: After presenting the fast-killing assay results, we now explicitly state that all subsequent infection experiments in the manuscript were performed using the slow-killing model. The revised manuscript reads (page – 5, lines – 131-134): "*We found that cmtr-1(jsn21) mutants exhibited significantly enhanced resistance to P. aeruginosa infection under both fast-killing (Fig S2A) and slow-killing conditions (Fig 2A). For the remainder of the study, we focused on the slow-killing model to investigate P. aeruginosa pathogenesis.*"

Comment: 4. Lines 134-139: The authors mention that it was unexpected for pan-tissue expression of cmtr-1 did not restore colonization of PA14 but it did however suppress enhanced survival. Could the authors comment on why this may be? Maybe in the discussion section?

Response: We have added the possible explanation. The revised manuscript reads (pages – 5-6, lines – 142-150): "*Notably, intestine-specific expression of cmtr-1 restored intestinal colonization, whereas pan-tissue expression failed to do so (Fig 2D, 2E). This observation was unexpected because both rescue constructs effectively suppressed the enhanced survival phenotype. One possible explanation is that the expression pattern driven by the sur-5 promoter does not fully recapitulate endogenous cmtr-1 expression. Additionally, previous studies have demonstrated that reduced bacterial colonization and enhanced survival are not always directly correlated [7,12,28]. Nevertheless, these findings established that intestinal CMTR-1 is sufficient to restore colonization in cmtr-1(jsn21) mutants.*"

Comment: 5. Lines 142-147: The authors used kanamycin-killed E. coli for lifespan experiments. However, there have been multiple publications in the field identifying this as not the best way to make sure the bacteria are metabolically inactive (PMIDS: 33637830, 37746065). Could the authors expand on why they didn't use PFA? I would also recommend adding a comment about this in the methods section.

Response: We thank the reviewer for raising this important point. Although PFA treatment has been proposed as an alternative method to generate metabolically inactive bacteria, previous studies have shown that PFA-killed *E. coli* can affect multiple aspects of *C. elegans* physiology, including delayed development, reduced brood size, and altered lipid storage (PMID: 37746065). Kanamycin-killed *E. coli* has been used in recent studies as a source of metabolically inactive bacteria (PMID: 39147935). In our experiments, worms grown on kanamycin-killed *E. coli* did not exhibit obvious defects in development, brood size, or overall lipid levels. Because we did not directly test the PFA-based method in this study, we are unable to compare the two approaches experimentally.

Comment: 6. Lines 175-180: The authors state that there was a "severely reduced brood size" and animals being "sterile" but did not include data anywhere in the figures to support this.

Response: We have now added brood size data upon knockdown of *pmk-1* and *kgb-1* in *cmtr-1(jsn21)* worms. The data is shown in Figure S5A.

Comment: 7. Overall, all the fluorescent images could be brighter. In some cases, it is difficult to see the change in expression by eye. It is acknowledged that the authors do include the quantification of GFP levels in all cases, and this is appreciated.
Response: To facilitate visualization and identification of worms in the fluorescence images, we have added dotted outlines marking worm boundaries in all relevant figures.
