# Supplementary material for: RNA methyltransferase CMTR-1 inhibition activates a GATA transcription factor-mediated protective immune response
Source: PLoS Pathog. 2026 Jun 24;22(6):e1014375. doi: 10.1371/journal.ppat.1014375 (PMC13318185; doi:10.1371/journal.ppat.1014375)
Supplement: Peer review history

# Peer review: Decision Letter 0

**DOI:** 10.1371/journal.ppat.1014375.r001

**Authors:** Read Pukkila-Worley, Eva Heinz

30 Mar 2026

PPATHOGENS-D-26-00098

RNA methyltransferase CMTR-1 inhibition activates a GATA transcription factor-mediated protective immune response

PLOS Pathogens

Dear Jogender,

Looking at the Editorial log in PLOS Pathogens, I can see that your paper had quite a journey from when you submitted it back in January! I was asked to be Academic Editor for your paper on March 9, and fortunately, the process unfolded very quickly from there. Your manuscript was evaluated by three expert reviewers in our field. You will see from their comments that the reviewers were overall quite positive about your manuscript and think it will likely be suitable for publication in PLOS Pathogens, after a few issues are addressed. Where possible, I encourage you do address their major critiques with new data, as I think some important points have been raised that will likely be shared by other scientists.

I am happy to serve as Academic Editor on the revision, so please direct it to me in your cover letter and in Editorial Manager when you resubmit your manuscript.

Please submit your revised manuscript by May 29 2026 11:59PM. If you will need more time than this to complete your revisions, please reply to this message or contact the journal office at plospathogens@plos.org. When you're ready to submit your revision, log on to https://www.editorialmanager.com/ppathogens/ and select the 'Submissions Needing Revision' folder to locate your manuscript file.

Please include the following items when submitting your revised manuscript:

* A letter that responds to each point raised by the editor and reviewer(s). You should upload this letter as a separate file labeled 'Response to Reviewers'. This file does not need to include responses to any formatting updates and technical items listed in the 'Journal Requirements' section below.

* A marked-up copy of your manuscript that highlights changes made to the original version. You should upload this as a separate file labeled 'Revised Manuscript with Track Changes'.

* An unmarked version of your revised paper without tracked changes. You should upload this as a separate file labeled 'Manuscript'.

If you would like to make changes to your financial disclosure, competing interests statement, or data availability statement, please make these updates within the submission form at the time of resubmission. Guidelines for resubmitting your figure files are available below the reviewer comments at the end of this letter.

We look forward to receiving your revised manuscript.

Kind regards,

Read Pukkila-Worley, M.D.

Academic Editor

PLOS Pathogens

Eva Heinz

Section Editor

PLOS Pathogens

Sumita Bhaduri-McIntosh

Editor-in-Chief

PLOS Pathogens

orcid.org/0000-0003-2946-9497

Michael Malim

-->Editor-in-Chief

PLOS Pathogens

orcid.org/0000-0002-7699-2064

**Journal Requirements:**

If the reviewer comments include a recommendation to cite specific previously published works, please review and evaluate these publications to determine whether they are relevant and should be cited. There is no requirement to cite these works unless the editor has indicated otherwise.

1) Please ensure that the CRediT author contributions listed for every co-author are completed accurately and in full.

At this stage, the following Authors/Authors require contributions: Annesha Ghosh, and Jogender Singh. Please ensure that the full contributions of each author are acknowledged in the "Add/Edit/Remove Authors" section of our submission form.

The list of CRediT author contributions may be found here: https://journals.plos.org/plospathogens/s/authorship#loc-author-contributions

2) We ask that a manuscript source file is provided at Revision. Please upload your manuscript file as a .doc, .docx, .rtf or .tex. If you are providing a .tex file, please upload it under the item type u2018LaTeX Source Fileu2019 and leave your .pdf version as the item type u2018Manuscriptu2019.

3) Please upload all main figures as separate Figure files in .tif or .eps format. For more information about how to convert and format your figure files please see our guidelines:

https://journals.plos.org/plospathogens/s/figures

4) We notice that your supplementary Figures are included in the manuscript file. Please remove them and upload them with the file type 'Supporting Information'. Please ensure that each Supporting Information file has a legend listed in the manuscript after the references list.

5) In the online submission form, you indicated that your data will be submitted to a repository upon acceptance. We strongly recommend all authors deposit their data before acceptance, as the process can be lengthy and hold up publication timelines. Please note that, though access restrictions are acceptable now, your entire minimal dataset will need to be made freely accessible if your manuscript is accepted for publication. This policy applies to all data except where public deposition would breach compliance with the protocol approved by your research ethics board. If you are unable to adhere to our open data policy, please kindly revise your statement to explain your reasoning and we will seek the editor's input on an exemption.

6) Please amend your detailed Financial Disclosure statement. This is published with the article. It must therefore be completed in full sentences and contain the exact wording you wish to be published.

1) State the initials, alongside each funding source, of each author to receive each grant. For example: "This work was supported by the National Institutes of Health (####### to AM; ###### to CJ) and the National Science Foundation (###### to AM)."

2) State what role the funders took in the study. If the funders had no role in your study, please state: "The funders had no role in study design, data collection and analysis, decision to publish, or preparation of the manuscript."

3) If any authors received a salary from any of your funders, please state which authors and which funders..

If you did not receive any funding for this study, please simply state: u201cThe authors received no specific funding for this work.u201d

7) 1) Some material included in your submission may be copyrighted. According to PLOS's copyright policy, authors who use figures or other material (e.g., graphics, clipart, maps) from another author or copyright holder must demonstrate or obtain permission to publish this material under the Creative Commons Attribution 4.0 International (CC BY 4.0) License used by PLOS journals. Please closely review the details of PLOS's copyright requirements here: PLOS Licenses and Copyright. If you need to request permissions from a copyright holder, you may use the PLOS Content Copyright Permission Form Please respond directly to this email and provide any known details concerning your material's license terms and permissions required for reuse, even if you have not yet obtained copyright permissions or are unsure of your material's copyright compatibility. Once you have responded and addressed all other outstanding technical requirements, you may resubmit your manuscript within Editorial Manager. Potential Copyright Issues:

i) Figures 1A, 5A, and S1C. Please confirm whether you drew the images / clip-art within the figure panels by hand. If you did not draw the images, please provide (a) a link to the source of the images or icons and their license / terms of use; or (b) written permission from the copyright holder to publish the images or icons under our CC BY 4.0 license. Alternatively, you may replace the images with open source alternatives. See these open source resources you may use to replace images / clip-art: - https://commons.wikimedia.org - https://openclipart.org/.

**Reviewers' Comments:**

Reviewer's Responses to Questions

**Part I - Summary**

Please use this section to discuss strengths/weaknesses of study, novelty/significance, general execution and scholarship.

Reviewer #1: The authors found that a forward genetic screen using a clec-60p::gfp reporter identified a hypomorphic allele of the mRNA cap methyltransferase CMTR-1 that activates basal immune signaling in C. elegans. Transcriptomic analysis showed broad induction of innate immune genes in cmtr-1(jsn21) mutants, leading to enhanced resistance to PA14. A transcription factor RNAi screen and bioinformatic analyses identified the intestinal GATA factor ELT-2 as the key regulator of this response, indicating that disruption of CMTR-1–mediated mRNA cap modification triggers a protective immune program.

Overall, this study identifies CMTR-1–mediated mRNA cap modification as a potential regulator of basal immune activation in C. elegans and provides evidence that disruption of this pathway induces a broad innate immune program that enhances resistance to PA14 infection. The genetic screen, transcriptomic analyses, and transcription factor RNAi screen collectively suggest that the intestinal GATA transcription factor ELT-2 plays an important role in mediating this response. However, several conclusions, particularly those regarding translation-independent immune activation and independence from major immune pathways, require additional experimental support. Addressing these concerns through further genetic epistasis analyses and mechanistic experiments would strengthen the authors’ claims and clarify the positioning of CMTR-1 within established immune signaling pathways. With these revisions, the study has the potential to provide meaningful insight into how perturbations in mRNA cap modification can act as a signal to activate protective immune responses.

Reviewer #2: The authors seek to discover novel regulators of the innate immune response in C. elegans and perform a forward genetic screen to identify mutations that upregulate the immune response gene clec-60. They confirm that G126E missense mutation in the mRNA cap1 methyltransferase cmtr-1 induces clec-60 expression in a recessive manner. Concomitantly, the authors observe that cmtr-1(G126E) confers resistance to PA14 infection and prevents intestinal accumulation of PA14-GFP. cmtr-1 is an essential gene required for the translation of all capped mRNAs, so to understand whether this mutation specifically activates the immune response the authors perform RNA sequencing. They find that the most prominently upgoing genes are indeed annotated as immune response genes. Using a candidate approach, the authors observe that induction of clec-60 by cmtr-1(G126E) is independent of canonical immune response pathways mediated by ZIP-2, DBL-1, and HLH-30. Finally, the authors identify the transcription factor ELT-2 as being required for the upregulation of clec-60 by cmtr-1(G126E) and the resulting resistance to PA14 infection. Together the findings uncover a novel regulator of the C. elegans immune response and open up interesting future directions such as (1) what is the mechanism for how cmtr-1(G126E) alters gene expression of ELT-2 targets, and (2) are there physiological contexts in which CMTR-1 activates the immune response? In general, the experiments are carefully done and the manuscript is clearly written. However some of their conclusions need rewording or additional support before publication, which I support.

Reviewer #3: Here the authors identify a knowledge gap in the field that can be alleviated through systemic perturbations to uncover endogenous pathways that enhance pathogen resistance using clec-60p::gfp reporter expression as a readout. With the use of an EMS screen in C. elegans, they identify a recessive mutation in cmtr-1 causing expression to be high without the presences of a pathogen. Through further verification, they find that this loss-of-function mutation enhances resistance to the pathogen Pseudomonas aeruginosa in slow-kill assays, corresponding to reduced intestinal accumulation of PA14. To understand if the cmtr-1lf mutant was exerting a broad innate immunity transcriptional response, they performed RNAseq and found innate immune genes were upregulated while the translation-related genes were downregulated. To determine if the enhanced immunity was due to translation inhibition, they examined clec-60p::gfp expression in zip-2 mutants with the cmtr-1 mutant background and found immune activation occurs independently from ZIP-2. Using GO term enrichment analysis and HOMER motif enrichment, the authors identified the ELT-2 binding motif as enriched, ELT-2 functions downstream of cmtr-1(jsn21) and is required for the enhanced pathogen resistance. Overall, this work provides an interesting and unique perspective in the field of immune activation and pathogenicity.

**********

**Part II – Major Issues: Key Experiments Required for Acceptance**

Please use this section to detail the key new experiments or modifications of existing experiments that should be absolutely required to validate study conclusions.

Generally, there should be no more than 3 such required experiments or major modifications for a "Major Revision" recommendation. If more than 3 experiments are necessary to validate the study conclusions, then you are encouraged to recommend "Reject".

Reviewer #1: 1) cmtr-1 mutants promoting PA14 resistance independent of translation inhibition: I think the authors need to analyse further before making such a bold conclusion.

Here, the authors only tested the role of ZIP-2 in contributing to PA14 resistance. A better strategy would be to analyse clec-60::gfp or PA14 resistance following RNAi inhibition of protein translation-related genes that are reduced in the cmtr-1 mutants.

2) cmtr-1 mutants promoting PA14 resistance independent of major immune pathways.

I am not convinced that the authors carried out enough genetic epistasis experiments to conclude this. Although generating kgb-1 and pmk-1 double mutants with cmtr-1 was not possible, the authors could use RNAi to inhibit kgb-1 or pmk-1 in the cmtr-1 for two generations. Alternatively, they could perform cmtr-1(RNAi) in the kgb-1 and pmk-1 mutants and assess survival on PA14. clec-60 qPCR can also be done.

3) elt-2(RNAi) suppresses the PA14 resistance of cmtr-1 mutants to the level same as elt-2(RNAi) alone. This suggests that elt-2 is required for survivability on PA14 independent of any upstream perturbations. Authors should use ELT-2::GFP and check for nuclear localization in the absence of cmtr-1. This will provide more support for ELT-2 functioning downstream of CMTR-1.

Reviewer #2: 1. In the section beginning on line 164 and the title to Figure 4 the authors claim, “Enhanced immunity in cmtr-1(jsn21) is independent of translation inhibition”. I do not think the data is the paper is sufficient to support this statement. The authors do convincingly show that enhanced immunity by cmtr-1 is independent of ZIP-2, but couldn’t translation inhibition still be mediating the cmtr-1 effect in a ZIP-2-independent manner? To support this statement the authors would need to inhibit translation through alternate means (there are many drugs and mutations that can do this) and show that this does not induce clec-60 in a zip-2 mutant. In lieu of these experiments, perhaps the authors would like to scale back their claims.

2. From genetic epistasis experiments the authors conclude that elt-2 acts downstream of cmtr-1(G126E) to mediate clec-60 expression. This is supported by elt-2 RNAi not affecting the low basal level of immune response genes via qPCR (Fig S4b). However in the more sensitive gfp reporter assay, elt-2 RNAi does lower the basal expression of clec-60::gfp (Figure 5d, p<0.001). Have the authors tested whether elt-2 is required for clec-60 induction by pathogen? If it is not required, that would support their claims that elt-2 acts downstream of cmtr-1, as opposed to being a general factor required for basal transcription of immune genes. Perhaps the authors would like to leave this open as a possible interpretation of their data.

Reviewer #3: 1. The effects of pathogens on host physiology can result due to the presence of the microbe or from the toxins that are secreted. In C. elegans, it is common to test pathogen resistance in the presence of fast-kill assays and slow-kill assays. This manuscript only includes slow-kill assays, and this reviewer would recommend strengthening their argument by adding in fast-kill assay data as well. The reviewer acknowledges that the two assays test two different things (death via colonization vs. death from produced toxins) but still thinks this assay should be included.

2. Lifespan assays were performed exclusively with 50 ug/mL FUdR. FUdR has many complex effects on aging phenotypes and many in the field believe that it is necessary to have at least one replicate of lifespan data without FUdR to support findings from the use with FUdR due to possible confounding effects. The authors should perform at least one replicate without FuDR for lifespan assays to confirm that the results are not due to FUdR and are due to the mutation in cmtr-1.

**********

**Part III – Minor Issues: Editorial and Data Presentation Modifications**

Please use this section for editorial suggestions as well as relatively minor modifications of existing data that would enhance clarity.

Reviewer #1: 1) Incidentally, an identical point mutation was independently isolated in a genetic screen for suppressors of hyperoxia sensitivity in gas-1(fc21) mutants (Meisel et al., 2024), where it displayed a dominant inheritance. (Line 103-105) is not useful.

2) Strikingly, while intestine-specific expression of cmtr-1 restored colonization, pan-tissue expression failed to do so (Figure 2D, E). This discrepancy was unexpected because both rescue constructs suppressed the enhanced survival phenotype. (Line 133-135)

Can authors do a tissue-specific knockdown (RNAi) using sensitive RNAi strains and determine the tissue specificity of CMTR-1?

3) Can authors please check the clec-60 mRNA levels in wildtype animals following PA14 exposure in control and elt-2(RNAi) to determine the requirement of elt-2 in promoting clec-60 expression?

4) Genotype nomenclature: please remove underscore (-) when writing for genotypes for two different genes. Instead, use a comma for RNAi and semi colon for double mutants.

Reviewer #2: 3. The authors should include some discussion of the effects G126E mutation may have on CMTR-1 activity. The cmtr-1(G126E) mutation lies in the G-patch domain of CMTR-1 (not the methyltransferase domain), which binds to the RNA helicase DDX-15 and regulates CMTR-1’s methyltransferase activity. Interestingly, disruption of the G-patch domain in vitro can either activate or inhibit CMTR-1 activity (see work from labs of Victoria Cowling, Janusz Bujnicki). I don’t think it’s necessary for the authors to attempt DDX-15 RNAi in their clec-60::gfp strain, but some acknowledgement of the work done on CMTR-1’s G-patch domain would add to the scope of the paper and inform on potential mechanisms.

Reviewer #3: 1. Throughout the manuscript and within figures and figure legends the authors switch between the use of wild-type and N2. All mentions of N2 should be replaced with WT to prevent any confusion that may arise, especially with readers not familiar with C. elegans strains.

2. Most of the survival plots and lifespan plots don’t contain statistical information in the figure (only in the legends). It would be helpful to show stats on the figures as well.

3. Lines 124-129: When discussing improved pathogen resistance, authors should note in the main manuscript text that the assay performed was a slow-kill assay since both slow-kill and fast-kill assays are the standard to perform when measuring pathogen resistance.

4. Lines 134-139: The authors mention that it was unexpected for pan-tissue expression of cmtr-1 did not restore colonization of PA14 but it did however suppress enhanced survival. Could the authors comment on why this may be? Maybe in the discussion section?

5. Lines 142-147: The authors used kanamycin-killed E. coli for lifespan experiments. However, there have been multiple publications in the field identifying this as not the best way to make sure the bacteria are metabolically inactive (PMIDS: 33637830, 37746065). Could the authors expand on why they didn’t use PFA? I would also recommend adding a comment about this in the methods section.

6. Lines 175-180: The authors state that there was a “severely reduced brood size” and animals being “sterile” but did not include data anywhere in the figures to support this.

7. Overall, all the fluorescent images could be brighter. In some cases, it is difficult to see the change in expression by eye. It is acknowledged that the authors do include the quantification of GFP levels in all cases, and this is appreciated.

**********

PLOS authors have the option to publish the peer review history of their article (what does this mean?). If published, this will include your full peer review and any attached files.

If you choose “no”, your identity will remain anonymous but your review may still be made public.

**Do you want your identity to be public for this peer review?**  For information about this choice, including consent withdrawal, please see our Privacy Policy.

Reviewer #1: No

Reviewer #2: No

Reviewer #3: No

**Figure resubmission:**

-->While revising your submission, we strongly recommend that you use PLOS’s NAAS tool (https://ngplosjournals.pagemajik.ai/artanalysis) to test your figure files. NAAS can convert your figure files to the TIFF file type and meet basic requirements (such as print size, resolution), or provide you with a report on issues that do not meet our requirements and that NAAS cannot fix.

After uploading your figures to PLOS’s NAAS tool - https://ngplosjournals.pagemajik.ai/artanalysis, NAAS will process the files provided and display the results in the "Uploaded Files" section of the page as the processing is complete. If the uploaded figures meet our requirements (or NAAS is able to fix the files to meet our requirements), the figure will be marked as "fixed" above. If NAAS is unable to fix the files, a red "failed" label will appear above. When NAAS has confirmed that the figure files meet our requirements, please download the file via the download option, and include these NAAS processed figure files when submitting your revised manuscript.

**Reproducibility:**

To enhance the reproducibility of your results, we recommend that authors of applicable studies deposit laboratory protocols in protocols.io, where a protocol can be assigned its own identifier (DOI) such that it can be cited independently in the future. Additionally, PLOS ONE offers an option to publish peer-reviewed clinical study protocols. Read more information on sharing protocols at https://plos.org/protocols?utm_medium=editorial-email&utm_source=authorletters&utm_campaign=protocols

# Author comment: Author response to Decision Letter 1

**DOI:** 10.1371/journal.ppat.1014375.r002

13 May 2026

Attachment Submitted filename: Response to Reviewers.pdf

# Peer review: Decision Letter 1

**DOI:** 10.1371/journal.ppat.1014375.r003

**Authors:** D. Scott Samuels, Read Pukkila-Worley

12 Jun 2026

Dear Dr. Singh,

We are pleased to inform you that your manuscript 'RNA methyltransferase CMTR-1 inhibition activates a GATA transcription factor-mediated protective immune response' has been provisionally accepted for publication in PLOS Pathogens.

One note for you to consider when preparing the final version of your manuscript.  In the first version of the paper you noted that the exact same cmtr-1(G126E) mutation was isolated in a screen for enhanced mitochondrial health (Meisel et al, Current Biology 2024). As you noted then, the G126E mutation confers a dominant mitochondrial health phenotype, but a recessive immune response activation phenotype. These comparisons are very interesting, as it reveals how the exact same missense mutation in cmtr-1 can diverse effects on animal physiology. During the revisions, a Reviewer requested that this sentence be removed, so you deleted it. Another reviewer of your paper feels that this should be included.  I agree that the addition of this information is important to put your study in context with what has previously been published.  I request that you add this information back to the final version of your paper.

Before your manuscript can be formally accepted you will need to complete some formatting changes, which you will receive in a follow up email. A member of our team will be in touch with a set of requests.

Please note that your manuscript will not be scheduled for publication until you have made the required changes, so a swift response is appreciated.

IMPORTANT: The editorial review process is now complete. PLOS will only permit corrections to spelling, formatting or significant scientific errors from this point onwards. Requests for major changes, or any which affect the scientific understanding of your work, will cause delays to the publication date of your manuscript.

Should you, your institution's press office or the journal office choose to press release your paper, you will automatically be opted out of early publication. We ask that you notify us now if you or your institution is planning to press release the article. All press must be co-ordinated with PLOS.

Thank you again for supporting Open Access publishing; we are looking forward to publishing your work in PLOS Pathogens.

Best regards,

Read Pukkila-Worley, M.D.

Academic Editor

PLOS Pathogens

Eva Heinz

Section Editor

PLOS Pathogens

Sumita Bhaduri-McIntosh

Editor-in-Chief

PLOS Pathogens

orcid.org/0000-0003-2946-9497

Michael Malim

Editor-in-Chief

PLOS Pathogens

orcid.org/0000-0002-7699-2064

***********************************************************

Reviewer Comments (if any, and for reference):

Reviewer's Responses to Questions

**Part I - Summary**

Please use this section to discuss strengths/weaknesses of study, novelty/significance, general execution and scholarship.

Reviewer #1: (No Response)

Reviewer #2: The authors seek to discover novel regulators of the innate immune response in C. elegans and perform a forward genetic screen to identify mutations that upregulate the immune response gene clec-60. They confirm that G126E missense mutation in the mRNA cap1 methyltransferase cmtr-1 induces clec-60 expression in a recessive manner. Concomitantly, the authors observe that cmtr-1(G126E) confers resistance to PA14 infection and prevents intestinal accumulation of PA14-GFP. cmtr-1 is an essential gene required for the translation of all capped mRNAs, so to understand whether this mutation specifically activates the immune response the authors perform RNA sequencing. They find that the most prominently upgoing genes are indeed annotated as immune response genes. They also find that translation-related genes are down-regulated, and through RNAi experiments show that their cmtr-1 mutation may enhance the immune response via translation inhibition. However, using a candidate approach, the authors observe that induction of clec-60 by cmtr-1(G126E) is independent of canonical immune response pathways mediated by ZIP-2, DBL-1, and HLH-30. Finally, the authors identify the transcription factor ELT-2 as being required for the upregulation of clec-60 by cmtr-1(G126E) and the resulting resistance to PA14 infection. Together the findings uncover a novel regulator of the C. elegans immune response and open up interesting future directions such as (1) what is the mechanism for how cmtr-1(G126E) alters gene expression of ELT-2 targets, and (2) are there physiological contexts in which CMTR-1 activates the immune response?

Reviewer #3: Here the authors identify a knowledge gap in the field that can be alleviated through systemic perturbations to uncover endogenous pathways that enhance pathogen resistance using clec-60p::gfp reporter expression as a readout. With the use of an EMS screen in C. elegans, they identify a recessive mutation in cmtr-1 causing expression to be high without the presences of a pathogen. Through further verification, they find that this loss-of-function mutation enhances resistance to the pathogen Pseudomonas aeruginosa in slow-kill assays, corresponding to reduced intestinal accumulation of PA14. To understand if the cmtr-1lf mutant was exerting a broad innate immunity transcriptional response, they performed RNAseq and found innate immune genes were upregulated while the translation-related genes were downregulated. To determine if the enhanced immunity was due to translation inhibition, they examined clec-60p::gfp expression in zip-2 mutants with the cmtr-1 mutant background and found immune activation occurs independently from ZIP-2. Using GO term enrichment analysis and HOMER motif enrichment, the authors identified the ELT-2 binding motif as enriched, ELT-2 functions downstream of cmtr-1(jsn21) and is required for the enhanced pathogen resistance. Overall, this work provides an interesting and unique perspective in the field of immune activation and pathogenicity. The authors have addressed all original major and minor comments and thus, this manuscript should be accepted for publication in PLOS Pathogens.

**********

**Part II – Major Issues: Key Experiments Required for Acceptance**

Please use this section to detail the key new experiments or modifications of existing experiments that should be absolutely required to validate study conclusions.

Generally, there should be no more than 3 such required experiments or major modifications for a "Major Revision" recommendation. If more than 3 experiments are necessary to validate the study conclusions, then you are encouraged to recommend "Reject".

Reviewer #1: (No Response)

Reviewer #2: All major issues have been addressed. The authors successfully performed all suggested experiments and have reinterpreted their data accordingly. I support publication of this manuscript.

Reviewer #3: The authors have addressed all previous concerns.

**********

**Part III – Minor Issues: Editorial and Data Presentation Modifications**

Please use this section for editorial suggestions as well as relatively minor modifications of existing data that would enhance clarity.

Reviewer #1: (No Response)

Reviewer #2: No minor issues.

Reviewer #3: The authors have addressed all previous concerns.

**********

PLOS authors have the option to publish the peer review history of their article (what does this mean?). If published, this will include your full peer review and any attached files.

If you choose “no”, your identity will remain anonymous but your review may still be made public.

**Do you want your identity to be public for this peer review?**  For information about this choice, including consent withdrawal, please see our Privacy Policy.

Reviewer #1: No

Reviewer #2: No

Reviewer #3: No

# Editor report: Acceptance letter

**DOI:** 10.1371/journal.ppat.1014375.r004

**Authors:** D. Scott Samuels, Read Pukkila-Worley

Dear Dr. Singh,

We are delighted to inform you that your manuscript, "

RNA methyltransferase CMTR-1 inhibition activates a GATA transcription factor-mediated protective immune response," has been formally accepted for publication in PLOS Pathogens.

We have now passed your article onto the PLOS Production Department who will complete the rest of the pre-publication process. All authors will receive a confirmation email upon publication.

The corresponding author will soon be receiving a typeset proof for review, to ensure errors have not been introduced during production. Please review the PDF proof of your manuscript carefully, as this is the last chance to correct any scientific or type-setting errors. Please note that major changes, or those which affect the scientific understanding of the work, will likely cause delays to the publication date of your manuscript. Note: Proofs for Front Matter articles (Pearls, Reviews, Opinions, etc...) are generated on a different schedule and may not be made available as quickly.

Soon after your final files are uploaded, the early version of your manuscript, if you opted to have an early version of your article, will be published online. The date of the early version will be your article's publication date. The final article will be published to the same URL, and all versions of the paper will be accessible to readers.

For Research Articles, you will receive an invoice from PLOS for your publication fee after your manuscript has reached the completed accept phase. If you receive an email requesting payment before acceptance or for any other service, this may be a phishing scheme. Learn how to identify phishing emails and protect your accounts at https://explore.plos.org/phishing.

Thank you again for supporting open-access publishing; we are looking forward to publishing your work in PLOS Pathogens.

Best regards,

Sumita Bhaduri-McIntosh

Editor-in-Chief

PLOS Pathogens

orcid.org/0000-0003-2946-9497

Michael Malim

Editor-in-Chief

PLOS Pathogens

orcid.org/0000-0002-7699-2064
